# Whole exome sequencing as a screening tool in dogs: A pilot study

**DOI:** 10.1016/j.csbj.2025.03.008

**Published:** 2025-03-07

**Authors:** Fréderique Boeykens, Evelien Bogaerts, Liesbeth Vossaert, Luc Peelman, Filip Van Nieuwerburgh, Jimmy H. Saunders, Bart J.G. Broeckx

**Affiliations:** a Laboratory of Animal Genetics, Department of Veterinary and Biosciences, Faculty of Veterinary Medicine, Ghent University, Merelbeke, Belgium; b Department of Morphology, Imaging, Orthopedics, Rehabilitation and Nutrition, Faculty of Veterinary Medicine, Ghent University, Merelbeke, Belgium; c Baylor College of Medicine, Department of Molecular and Human Genetics, Houston, TX United States; d Laboratory of Pharmaceutical Biotechnology, Faculty of Pharmaceutical Sciences, Ghent University, Ghent, Belgium; e Centre for Clinical Genetics of Companion Animals, Faculty of Veterinary Medicine, Ghent University, Merelbeke, Belgium

**Keywords:** Whole Exome Sequencing (WES), Canine genetics, Veterinary diagnostics, Genetic, Screening, Variant detection, Veterinary clinical genetics, Diagnostic tool

## Abstract

**Background:**

Whole-exome sequencing (WES) is used to selectively sequence all exons of protein-coding genes. WES is considered as a cost-effective and direct approach for identifying phenotype-associated variants in protein-coding regions and is as such situated between the traditional Sanger sequencing and whole genome sequencing (WGS). While WES is already widely used as a clinical tool in human and medical genetics, its use in veterinary medicine is currently restricted to research purposes. In this article, we aimed to provide baseline performance characteristics of a WES design to assess its suitability with future applications in veterinary clinical genetics in mind.

**Methods:**

To assess the potential of WES in a clinical setting for dogs, 49 canine samples underwent capture, sequencing and analysis for the presence of 352 known phenotype-associated variants. The sequencing performance was compared for three types of variants, based on their size and location: single nucleotide variants (SNVs) inside exons, larger indel variants (≤20 bp) inside exons and intronic variants.

**Results:**

On average, 85 % and 82 % of the exonic SNPs and larger variants were sequenced at a sequencing depth of ≥ 10x in the 49 samples, respectively. In the best performing sample, 94 % of the exonic SNPs were covered at least 10x, whereas in the worst performing sample, still 71 % of the exonic SNPs had an average sequencing depth of more than 10x.

**Conclusion:**

To our knowledge, this is the first report that describes the performance of a research-intended WES design if it would be used in clinical genetics. This study found that WES demonstrated high efficacy in detecting variants located within target regions, including those that were not initially included in the design. However, the performance varied across different variants. The next steps would be the development of improved designs and settings to ameliorate the results.

## Graphical Abstract

**Figure:**
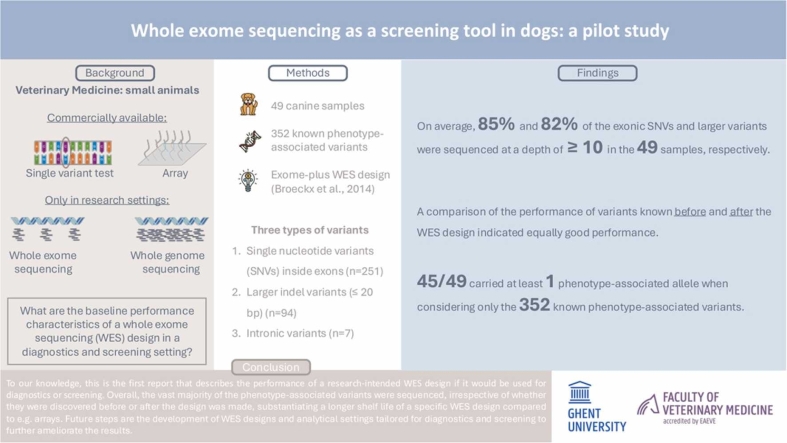

## 1 Introduction

Whole exome sequencing (WES) allows the selective sequencing of (protein-)coding regions in the genome [1]. A decade ago, WES was first used successfully to identify genetic variants responsible for Miller syndrome in humans [2]. Since then, WES has been used extensively to identify novel phenotype-associated variants, not only in humans but also in other species [3], [4]. As a result, WES designs are currently available for various species (e.g. mice, pigs and the dog) [5], [6], [1]. While the target regions tend to vary between designs, WES is generally considered to be a direct and cost-efficient approach to identify phenotype-associated variants in protein-coding regions and is as such situated between the traditional (low-throughput) Sanger sequencing and whole genome sequencing (WGS) approaches.

**Fig. 1:**
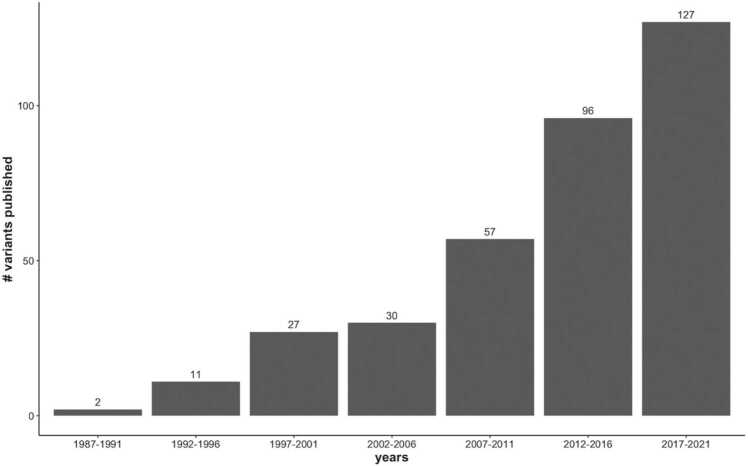
A representation of the number of new canine variants published per 5 years, starting from 1987 to 2021, based on the Online Mendelian Inheritance in Animals (OMIA) website.

In human clinical genetics, WES and WGS are used standardly in a diagnostic setting, side-by-side to single-gene tests and genotyping arrays and each of these techniques has its specific application. Single-gene tests are for example ideal in case of a very specific differential diagnosis, with pathognomonic symptoms and no locus heterogeneity. These diseases represent, however, only a small subset of all genetic diseases in humans. In case of a broader differential, and for diseases with higher locus heterogeneity, single-gene tests rapidly become cost-inefficient, neither do they sufficiently often lead to a diagnosis and time-consuming cascade testing has to be done. While genotyping arrays are cost-efficient for screening large sets of variants simultaneously, the downside is that their design is fixed. Practically, this implies that only variants that were included in the design stage, will be genotyped, and every variant discovered after design, will not be covered. At the current discovery rate (Fig. 1), this also implies that arrays have to be redeveloped at an enormous pace and are continuously lagging behind. In contrast, WES and WGS offer more comprehensive genetic analysis. Although they are more expensive, they can identify both known and novel disease-causing variants. Furthermore, various studies demonstrated that their diagnostic yield was substantially higher than standard single gene tests or chromosomal microarrays in more demanding cases (e.g. those with locus heterogeneity) [7], [8], [9]. Since WGS is not yet widely implemented in clinical settings, direct comparisons between WES and WGS remain limited. One study found that diagnostic rates of WES and WGS appeared to be comparable, even though the exome contains only 1–2 % of the genome [10], while others report an incremental yield when using WGS. It is estimated that 80–85 % of all known human disease-causing variants are located in the exonic regions, but WGS does enable a better detection of non-coding variants, copy number changes, and even short tandem repeat expansions and mitochondrial DNA variants. Either way, the cost effectiveness and increased diagnostic yield compared to single gene and panel testing have resulted in the extensive use of WES in human clinical genetics [4], [3].

Although WES has been widely used for over a decade, its application in veterinary medicine remains limited. WES is currently only used for research purposes in veterinary medicine and not as a screening or diagnostics tool [11], [12], [13], [14], [15]. For screening and diagnostics, most of the large companies in the veterinary market use array-based genotyping methods, while some use single-gene tests [16], thus most currently available data is mainly array-based. While high average call rates and a high reproducibility are for example promised by the manufacturers of arrays, there is no knowledge on average call rates of WES, so a direct comparison is currently not possible [17]. In theory, WES should be capable of detecting most newly discovered variants after its initial development, giving it a long shelf-life. However, no data currently supports this assumption.

This study aims to evaluate the performance of a WES design for potential use in veterinary screening, with a focus on future applications in veterinary clinical genetics [18]. This implies firstly that variants previously found to be associated with phenotypes have to be detected. This requires a sufficiently high and consistent sequencing depth of the regions of interest. This will depend on the location of the variants, i.e. whether they are located in- or outside the coding sequences (CDS), and thus on whether those regions are captured within the WES library design. Secondly, we evaluated the detection not only of variants that were known at the time of the development of the WES library, but also variants discovered afterwards. As a final step, a proof-of-concept and a demonstration of the potential application of WES in a screening setting, all WES sequenced samples are investigated simultaneously for the presence of the largest set of canine phenotype-associated variants (>350 variants) reported in literature so far, to demonstrate that 1/ WES allows the simultaneous detection of multiple variants and 2/ that animals in general and dogs more specifically may carry several disease-associated variants even though they do not show symptoms themselves, which is key for breeding advice and thus veterinary clinical genetics. In this study, WES was used as a general screening tool to explore its potential in veterinary clinical genetics and none of the dogs had clinical symptoms associated with the disease-associated variants tested. By assessing a set of known phenotype-associated variants, we aim to understand how WES could be applied in practice and contribute to bridging the gap between genetic research and clinical veterinary care.

## 2 Materials and methods

### 2.1 Samples

EDTA-blood samples were collected for research purposes. Approval was granted by the local ethical (Faculty of Veterinary Medicine, Ghent University, Belgium) and deontological (Federal Public Service Health, Food Chain Safety and Environment, Brussels, Belgium) committees (EC2017/86). All experiments were carried out in accordance with the approved guidelines. The performance evaluation of WES was based on 49 dogs from different dog breeds, including sixteen Labrador retrievers (male: 7; female: 9), seven Deerhounds (male: 2; female: 5), four Malinois (male: 3; female: 1), five Bloodhounds (male: 1; female: 4), five crossbreeds (male: 3; female: 2), four Dutch partridge dogs (male: 1; female: 3) and one of each of the following breeds: Belgian shepherd (male: 1), English cocker spaniel (female: 1), Pitbull (male: 1), Irish setter (female: 1), American Staffordshire (male: 1), Chesapeake Bay retriever (male: 1), French bulldog (male: 1) and Jack Russel terrier (female: 1). Overall, 22 dogs were male, while 27 dogs were female. Age ranged from 11 weeks to 12 years. None of the dogs exhibited clinical symptoms associated with the disease-associated variants tested.

Genomic DNA was extracted with the DNeasy Blood & Tissue Kit (QIAGEN) with 100 μl of blood as input. The standard protocol was followed (including the RNAse A step) with the exception of the final elution step: instead of using 200 μ l of Buffer AE, only 100 μ l was used. Whole exome sequencing (WES) followed the method of Broeckx et al. [19].

Briefly, extracted DNA was fragmented on a Covaris S2 System, samples were end repaired, A-tailed and ligated with TruSeq adapters using the reagents from the NEBNext Ultra DNA Library Prep Master mix set for Illumina (New England Biolabs) according to the manufacturer’s protocol. Size selection was performed on a 2 % E-Gel (Invitrogen Life Technologies), fragments were selected with an insert size around 300 bp. One μl of the ligated product was subsequently amplified in an enrichment PCR (10 cycles) for library quality assessment as recommended in the ‘SeqCap EZ Library SR User’s Guide’ (Nimblegen, Roche). Thereafter, the pre-capture LM-PCR was performed on the samples for 8 cycles as prescribed in the SeqCap EZ library protocol. The concentration of each PCR product was determined using Quant-iTTM Picogreen® dsDNA Assay (Life Technologies). Each time four samples were equimolarly pooled to obtain a total DNA input of 1250 ng. The pooled library was hybridized for 67–68 hours with the baits (SeqCap Developer Library). The hybridized library was washed and the captured and pooled DNA was recovered. After a final amplification (LM-PCR, 18 cycles), the quality of the library was checked using the High Sensitivity DNA chip (Agilent). To check the fold enrichment after capturing, a qPCR is performed as a quality control step before sequencing. Five primer pairs were used, as described previously. An additional qPCR was performed to determine the quantity of the library to ensure optimal cluster densities. Each pool was sequenced in a separate run on the NextSeq 500 PE 75 bp.

### 2.2 WES and data analysis

WES was conducted using the “exome-plus” design as described in Broeckx et al. [19]. Reads were aligned to the CanFam3.1 reference genome using BWA version 0.7.17 [20]. Duplicate reads were marked with MarkDuplicatesSpark tool (GATK/4.1.8.1-GCCcore-9.3.0-Java-1.8). Variant calling followed the GATK Best Practices pipeline (GATK version 4.1.8.1), and only variants that passed the “hard” quality filter recommended by GATK Best Practices were retained from the total list of putative variants in the.vcf file [4].

### 2.3 List of phenotype-associated variants

A list of phenotype-associated variants was downloaded from the Online Mendelian Inheritance in Animals (OMIA) website (https://www.omia.org/). Only variants that met the following four criteria were included in the analysis: 1/ The variant had a known genomic or mitochondrial location (**denoted as “g.XXX” or “m.XXX” in the file**). 2/ The variant was mapped to the **CanFam3.1 reference sequence**. 3/ The gene name was correctly annotated.

4/ The variant was **≤ 20 base pairs** (cutoff applied by OMIA for larger variants).

### 2.4 Analysis of sequencing depth at the loci where phenotype-associated variants reside

Sequencing depth analysis was performed separately for three variant categories: 1/ Single-nucleotide variants (1 bp) in coding sequences (CDS) of protein-coding genes, 2/ Small intronic and untranslated region (UTR)-associated variants (1 bp) and 3/ Larger variants (>1 bp and ≤20 bp). Among the seven intronic variants, four were located near exon-intron boundaries, impacting splicing mechanisms [21], [22], [23], [24]. Notably, one variant was situated within a promoter region [25] and the remaining two were classified as deep intronic variants, located 3 kb to 10 kb away from the nearest exon [26], [27].

Sequencing depth was evaluatd using three cutoffs: ≥ 10x, ≥ 20x and ≥ 30x. Depth values were calculated with the DepthOfCoverage function from the GATK version 4.1.8.1 and further processed with custom scripts in R version 4.1.1.

### 2.5 Phenotype-associated variants identified in the samples

The.vcf files were searched for the known phenotype-associated variants. Using an in-house developed R-package (variantscanR) [28]. General performance characteristics were described, as well as the detection of clinically relevant variants.

### 2.6 The association between the number of variants tested and the proportion of the population in which at least variant was present

To investigate how the number of dogs with at least one detected variant changes with the availability of more variants for screening, we randomly selected subsets of disease-associated variants that increased in size by 50 variants at each step.

## 3 Results

### 3.1 Description of the phenotype-associated variants

Of the 477 variants downloaded from OMIA, 63 were excluded due to missing or incorrect reference genome annotations or unspecified genomic/mitochondrial locations ("g." or "m."). The remaining 414 variants were manually curated, and those exceeding 20 bp in size or lacking a genomic location were excluded, resulting in a final set of 352 variants for downstream analysis (suppl. File 1.). Among these, 258 were single-nucleotide variants (SNVs), while 94 were larger variants (>1 bp and ≤20 bp). In the group of one basepair variants, 251 fell within the coding sequences (CDS) and 7 were intronic or were located in UTRs. These 352 variants were located in 242 different genes with a total of 798 unique transcripts.

### 3.2 Performance per phenotype-associated variant and per sample

The first analysis assessed sequencing performance across all 49 samples at the variant level. Sequencing depth varied considerably across variant categories. The mean sequencing depth for single-nucleotide CDS variants was 35.44x (range: 0x-290x). The mean sequencing depth for larger variants was 36.90, with minima and maxima of 0x and 341x, respectively. For the intronic/UTR loci, the mean sequencing depth per variant was markedly lower with a value of 5.17, with minima and maxima of 0x and 46x, respectively.

The second analysis evaluated sequencing performance at the sample level across all 352 variants (Table 1). In this analysis, a variant was only counted if it reached a certain sequencing threshold (i.e. ≥10x, ≥20x or ≥30x). Based on the data, several trends are visible. Firstly, for each category, the number of successfully sequenced loci decreases with increasing sequencing depth thresholds. Secondly, the highest percentage of successfully sequenced loci was found in the one basepair protein coding (CDS) category, followed closely by the larger variants category and finally the intronic/UTR loci.

**Table 1:** **Per sample performance:** minimum, maximum, median and mean number of variants detected per sample at ≥ 10x, ≥ 20x and ≥ 30x sequencing depth for intronic and exonic one basepair variants and larger variants (≤ 20 bp).

Intronic (n = 7)								
	min		max		median		mean	
	#	%	#	%	#	%	#	%
≥ 10	0	0	2	28.57	1	14.29	1.43	20.43
≥ 20	0	0	1	14.29	1	14.29	0.73	10.43
≥ 30	0	0	1	14.29	0	0	0.31	4.43

Exonic (n = 251)								
	min		max		median		mean	
	#	%	#	%	#	%	#	%
≥ 10	178	70.92	238	94.82	215	85.66	214.29	85.26
≥ 20	99	39.44	212	84.46	164	65.34	162.92	64.54
≥ 30	59	23.51	168	66.93	117	46.61	116.43	46.22

Larger variants (n = 94)								
	min		max		median		mean	
	#	%	#	%	#	%	#	%
≥ 10	67	71.28	89	94.68	78	82.98	78.31	82.98
≥ 20	37	39.36	73	77.66	60	63.83	58.86	61.70
≥ 30	27	28.72	63	67.02	41	43.62	41.37	43.62

# number of variants; %, percentage of variants

### 3.3 The probability to identify variants discovered after the designing process

To assess WES performance on newly discovered variants, we reanalyzed sequencing depth using only variants published after the design's development in 2014 [19], [1]. Since then, 170 variants were published including 6, 118 and 46 one bp intronic/UTR variant, one bp CDS variants and larger variants, respectively (Table 2).

**Table 2:** **Per sample performance of variant published after 2014:** minimum, maximum, median and mean number of variants detected per sample at ≥ 10x, ≥ 20x and ≥ 30x sequencing depth for intronic and exonic one basepair variants and larger variants (≤ 20 bp).

Intronic (n = 6)								
	min		max		median		mean	
	#	%	#	%	#	%	#	%
≥ 10	0	0	1	0.00	0	0.00	0,47	7.82
≥ 20	0	0	0	0.00	0	0.00	0	0.00
≥ 30	0	0	0	0.00	0	0.00	0	0.00

Exonic (n = 118)								
	min		max		median		mean	
	#	%	#	%	#	%	#	%
≥ 10	85	72.03	112	94.92	103	87.29	102.47	86.84
≥ 20	48	40.68	98	83.05	80	67.80	78.41	66.45
≥ 30	25	21.19	81	68.64	55	46.61	55.90	47.37

Larger variants (n = 46)								
	min		max		median		mean	
	#	%	#	%	#	%	#	%
≥ 10	33	71.74	42	91.30	38	82.61	37.65	82.85
≥ 20	20	43.48	36	78.26	29	63.04	28.53	62.02
≥ 30	15	32.61	30	65.22	22	47.83	21.35	46.41

When only looking at the one bp CDS variants described after the release of the WES design at a ≥ 10x sequencing depth, the median increased from 86 % to 87 %, the max remained at 95 % and the min value changed from 71 % to 72 %, respectively.

### 3.4 Actual phenotype-associated variants

As a proof-of-concept, all 49 samples were checked for the potential presence of any of these 352 phenotype-associated variants. Of these 352, a total of 23 different phenotype-associated variants were detected in these samples. Six variants were linked to coat color and type, including recessive black, long hair, black melanistic mask, coat color dilution, brown coat color, and red/yellow coat color [29], [30], [31], [32], [33], [34], two variants were related to the pharmacokinetics of a specific drug (i.e. metabolizer of a cognitive enhancer and adverse reaction to certain drugs) [35], [36], another one was related to litter size (fecundity) [37], one variant was associated with skull shape (brachycephaly) [38], and the remaining 13 variants were associated with diseases (i.e. Menkes disease [39], Wilson disease[39], Laryngeal paralysis and polyneuropathy [40], acral mutilation syndrome [27], skeletal dysplasia 2 [41], exercise-induced collapse [42], progressive rod-cone degeneration [43], leukodystrophy [44], degenerative myelopathy [45], von Willebrand disease [46], Stargardt disease 1 [47], upper airway syndrome [48], and neuronal ceroid lipofuscinosis 4 A [49].

The pattern of inheritance of these phenotypes is autosomal recessive for all except four of them, as black melanistic mask is autosomal dominant, Menkes disease is X-linked, leukodystrophy is mitochondrial and upper airway syndrome has a complex mode of inheritance.

Among the 49 dogs analyzed, 45 carried at least one mutant allele for a phenotype-associated variant (Table 3). A closer look at the subset of 13 disease-associated variants, revealed that 41 out of 49 dogs carried at least one allele associated with disease. Based on the coat-colour variants, genotype-phenotype accuracy was 100 %.

**Table 3:** The canine samples (n = 49) were checked for the number of different phenotype-associated variants and different disease associated variants. Δ variants column indicates the number of different variants found and does not include information regarding the zygosity status of these variants.

Δ Phenotype-associated variants	
Δ variants (n = 352)	Samples (n = 49)
0	4
1	0
2	4
3	10
4	10
5	18
6	1
7	2
Δ Disease-associated variants	
Δ variants (n = 323)	Samples (n = 49)
0	8
1	15
2	20
3	3
4	3

### 3.5 Discoveries of clinical relevance

Three male Labrador Retrievers and two male crossbreed dogs were hemizygous for the ATP7A variant associated with Menkes disease. Additionally, one Deerhound and one French Bulldog were homozygous for the ATP7B variant linked to Wilson disease [39]. Two dogs were homozygous for the variant in the *ADAMTS3* gene, associated with upper airway syndrome [48]. In 3 dogs, a mitochondrial variant in the *CYTB* gene was discovered. This variant is associated with canine spongiform leukoencephalomyelopathy [44]. Two Deerhounds were homozygous for the variant in the *ABCB1* gene. This variant is predominantly found in herding breed dogs and is associated with adverse effects following the administration of certain types of drugs [36]. One Dutch Partridge dog was genetically predisposed for a defective Von Willebrand factor plasma glycoprotein. The type I disease is characterized by low concentrations of the factor and leads to mild or moderate symptoms associated with coagulation issues [46]. Finally, one Bloodhound was homozygous for the mutant allele associated with canine degenerative myelopathy (DM) and is thus at risk for the development of DM at a later age [45].

### 3.6 Phenotypically healthy versus genetically free of disease

**Fig. 2:**
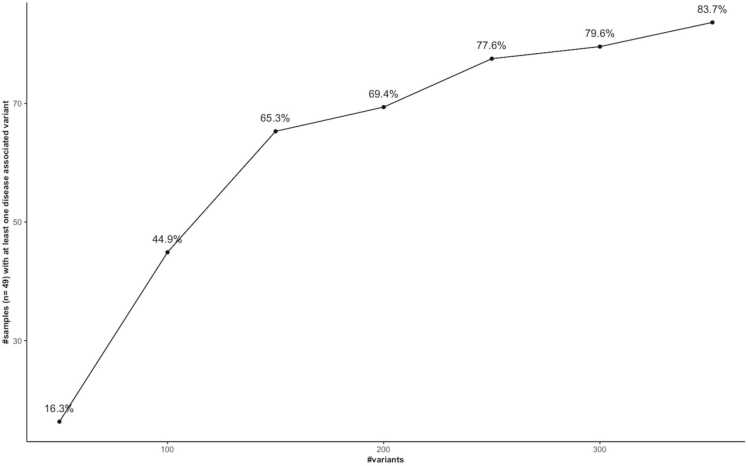
Proportion of dogs in which a disease-associated variant was detected for random subsets of variants of increasing size. Variant subset sizes: 50, 100, 150, 200, 250, 300, 323 (total variant set). Each subset was screened in the same population of dogs (n = 49).

Among the 49 samples, 16.3 % carried at least one phenotype-associated variant in the first subset of 50 randomly selected variants (Fig. 2). This increased to 44.9 %, 65.3 % and 69.4 % of the samples when looking at 100, 150 and 200 variants, respectively. At 300 variants, 79.6 % of the samples were carrier of at least one variant allele. The maximum number of samples carrying at least one variant was reached when screening for the entire disease-associated variant dataset and consisted of 41 out of 49 samples (83.7 %).

## 4 Discussion

In recent years, WES has been increasingly used in human medicine as a clinical tool to detect the underlying genetic cause of phenotypes that are difficult to diagnose [3]. As sequencing costs decrease, the application of WES as a clinical tool (i.e. used for diagnostics or screening purposes) in veterinary medicine becomes gradually more realistic. However, to our knowledge, literature on this topic is currently lacking. As such, we aimed to provide a first overview of the characteristics and clinical potential of a canine WES design that is frequently used.

WES primarily targets exons and exon-intron junctions, where most known phenotype-associated variants reside. As expected, our findings confirm that sequencing depth was higher for CDS variants (85.3 % ≥10x) compared to intronic/UTR variants (20.4 % ≥10x), ensuring reliable variant detection in protein-coding regions. For larger variants, only those where every single basepair part of the variant reached a certain minimum sequencing depth, were considered to be successfully sequenced. Intuitively, this makes it less likely for a large variant in comparison to one basepair variants to be successfully sequenced. Nevertheless, the sequencing results were in the same order of magnitude as the CDS variants (Table 1), which is of course very positive.

WES has the advantage that genotypes can be determined at every basepair that is sequenced at a sufficient sequencing depth. This also suggests that phenotype-associated variants discovered only after the WES design defining the target regions was made, might be identified. This is an important point to consider as discovery rates increase rapidly (Fig. 1). For example, in the period from 1987 to 1991 there were only 2 variants published, compared to the 127 in the period from 2017 to 2021, which is due to better detection methodologies, the availability of larger study populations, and more literature enabling a better interpretation of discovered variants.

Similar trends and percentages were obtained for one bp CDS and larger variants but not for intronic/UTR variants when looking at the one bp CDS variants before (including all variants) and after the release of the WES design at a ≥ 10x sequencing depth.

Based on our results, the probability of correctly sequencing and interpreting these new variants actually appears to be unchanged relative to the predesign published variants. This confirms the hypothesis that the vast majority of variants found after a WES design was made, will still reliably be detected, especially when the variants are located in the protein-coding regions. The trends seen in the probability of capturing and sequencing newly discovered variants are also intuitive: variants that fall within the target regions are far more likely to be sequenced than variants outside these target regions. Nevertheless, the probability for the latter variants is not zero due to off-target sequencing. Unlike microarrays, which can only detect preselected variants, WES offers flexibility by capturing newly discovered variants, even outside the initial target regions due to off-target sequencing. While this reduces efficiency, it provides an advantage in identifying emerging phenotype-associated variants [50].

In clinical settings, it is thus most important that the already known phenotype-associated variants are adequately sequenced regardless of variant size. The current WES design, developed for research purposes nearly a decade ago, could be optimized for clinical use by refining target capture regions and minimizing off-target sequencing. Adjusting for newly discovered phenotype-associated variants could further enhance diagnostic performance. Overall, it seems likely that the characteristics of the WES research design used here can easily be further improved.

This brings us to the next point, which is the minimal sequencing depth one should aim for. Unfortunately, there is no consensus on the minimal sequencing depth necessary in veterinary clinical settings to accept that a genotyped variant is correct and not a sequencing error. Furthermore, what is necessary in terms of sequencing depth also depends on the type of variant, the region where it is located and whether the goal is to detect somatic variants in cancer or germline variants [19], [51]. In oncology for example, a higher coverage is necessary to detect somatic variants with a lower allele frequency [52]. It is clear that the choice for any arbitrary cut-offs will have major implications on the performance parameters.

As a final proof-of-concept, we looked whether we could actually identify phenotype-associated variants in our samples. In total, 23 different variants were detected, with 13 of these variants linked to a disease. Although none of the dogs had clinical symptoms related to any of these diseases when the samples were collected, three Labrador Retrievers and two cross breed dogs were hemizygously affected for Menkes disease due to a missense variant in the *ATP7A* gene. Two dogs were homozygous for the variant associated with Wilson disease (*ATP7B* gene). While the symptoms in humans are far more severe for these diseases, the identification of these variants might warrant clinical action, i.e. dietary changes to prevent copper-associated issues [16], [39]. One Bloodhound was at risk for canine DM (*SOD1* gene). DM is a degenerative disease with late onset of symptoms and has been previously described in this breed [53], [54]. Although a dog may be at risk for this disease, not all dogs will develop symtoms, which can be attributed to the incomplete penetrance of the phenotype and/or the late onset of symptoms [53]. One Jack Russel and 2 Labrador retrievers carried the mitochondrial variant associated with canine spongiform leukoecephalomyelopathy (*CYTB* gene), a disorder discovered in Australian cattledogs and Shetland Sheepdogs [44]. One Dutch partridge dog was homozygous for the variant associated with von Willebrand disease type I (VWF gene). The von Willebrand factor is a protein necessary for platelet adhesion and aggregation. Clinical features are characterized by an increased bleeding tendency, but may be variable. Due to the varying severity of the condition, affected dogs are sometimes only identified after surgery or trauma, when excessive bleeding is observed. Two dogs, one Bloodhound and one American Staffordshire Terrier, are homozygous for the variant in the *ADAMTS3* gene, associated with Upper Airway syndrome. In these breeds, the genotype-phenotype association has however not yet been established, which highlights an interesting point of consideration when mass screenings are performed, as discussed further below [48]. Two Deerhounds are homozygous for a variant in the *ABCB1* gene and one Bloodhound is homozygous for a variant allele of the *CYP1A2* gene. Both variants are related to the pharmacokinetics of drugs. The related phenotypes are observable only after administration of the respective medication [35], [36].

Carriers were detected for skeletal dysplasia 2 (caused by a missense variant within the COL11A2 gene which results in mild disproportionate dwarfism), exercise-induced collapse (caused by a missense variant within the DNM1 gene), progressive rod-cone degeneration (caused by a missense variant within the PRCD gene), stargardt disease (caused by a frameshift insertion in the ABCA4 gene) and neuronal ceroid lipofuscinosis 4 A (caused by a missense variant in the ARSG gene) [41], [42], [43]. Since these diseases are inherited in an autosomal recessive manner and all these dogs carry only one variant allele of the disease-causing variants, clinical symptoms are not expected. These results are however important for breeding.

As the number of phenotype-associated variants is increasing rapidly, a point will be reached where no dog is free of disease-causing variants. Even within this article where 49 samples were screened on only 352 variants, already 23 phenotype-associated variants were detected, 13 of which were associated with disease. While it is obvious that excluding all dogs that carry disease-associated variants will lead to massive population bottlenecks, with the associated negative consequences, this also means that practical breeding advice should be given when these type of (high throughput) tests are offered to the general public. In line with other general recommendations, key is avoiding that diseased animals are born [55], [56]. Practically, for autosomal recessive diseases, it is important not to rule out heterozygous carriers and if certain prerequisites are fulfilled, even homozygously affected dogs might be used for breeding but only when they are combined with dogs homozygous for the wild type allele(s) for that disease [56]. With this strategy, no clinically affected dogs will be born and the genetic diversity in a dog population will not be reduced further. For X-linked recessive diseases in male animals, the same exception is true. Unfortunately, wisely combining dogs for breeding is not an optimal strategy for autosomal dominant, X-linked dominant diseases (both sexes) and X-linked recessive diseases (in female dogs) as every combination involving hetero/hemi- or homozygously affected dogs can lead to clinically affected progeny [55].

Before implementing WES in a clinical setting, several important considerations have to be made. Firstly, in the development of clinical tests it is of utmost importance that the reportable range, the proportion of the clinical target for which reliable targets can be generated, is defined prior to launching and provided to the clinician to guide in the interpretation of the results [57]. Furthermore, as already mentioned, variants might be missed when they are not sufficiently sequenced and the loci for which the sequencing depth is too low should be summed up in the clinical report. Secondly, ideally, all variants discovered during sequencing should be put in a national and/or international database as it will aid clinicians and researchers to classify variants according to their pathogenicity [57]. Thirdly, there is a need for adequate tools for variant filtering and interpretation. Many variants are detected with WES, which makes it very difficult to distinguish causal variants involved in a disease from e.g. incidental findings or benign variants that are possibly in linkage disequilibrium with the causative variant [58], [59]. Cooper et al. (2011) even concluded that nowadays identification of variants is no longer the limiting step, but assessing the deleteriousness of these variants is the main challenge [60], especially with the advent of whole genome sequencing and the detection of more e.g. non-coding variants, etc. A recent human study re-analyzed what were initially considered to be severe disease-causing variants in newly sequenced individuals and 27 % of these variants were re-evaluated to be most likely common polymorphisms or lacked direct evidence for pathogenicity [61]. To reduce false positive findings, it is important to combine bioinformatic and experimental support as much as possible [59]. In addition, unexpected but medically relevant variants might be discovered during sequencing. In human medicine these secondary, unintended findings are seen in approximately 4–5 % of the patients [3]. As such, it is important that patients are informed prior to testing about this possibility and they should have the opportunity to decide whether they want to be informed about these results or not [3], [62], [63]. In dogs, similar situations can occur. For example, in this study, dogs genetically predisposed for Wilson disease and DM, were found. These disorders have a middle to late age-of-onset, respectively. If found at an early age, dietary changes for Wilson disease and physiotherapy for DM influence disease progression and overall quality of life [39], [45], [64]. This indicates that incidental findings can have a positive impact on the health and well-being. However, the psychological impact of this information on pet owners and the corresponding actions taken, should not be neglected. A recent case was reported were a life-ending decision in a dog was made, solely based on a DNA-test result [65]. While the owners undoubtedly did this to for the good of their pet, it is an open question whether the symptoms were actually caused by the genetic disease and not by a wide range of other diseases with therapeutical options [65]. This substantiates why careful reporting is essential, and adequate genetic counselling in veterinary medicine should become a standard part of veterinary care. These observations have also led to the foundation of the clinical genetics advice center for companion animals at the Faculty of Veterinary Medicine at Ghent University.

The final issue is linked to the detection of variants that are known to be associated with a phenotype, but in a breed where the genotype-phenotype association has not yet been confirmed. For example, in this study a bloodhound was found to be genetically predisposed for Wilson disease. This particular disease has not yet been described in this breed. The detection of a disease-related variant in a breed where it had not been reported, may represent phenotypic expansion, but nevertheless, it should be taken into account that the breed's genetic background may affect the association with the phenotype, as demonstrated in mice [66]. A more recent example, closer to the dog, was found in the cat were a likely pathogenic variant was found in the Duchenne muscular dystrophy (*DMD*) gene that most likely leads to a phenotype similar to adult-onset Becker-Type muscular dystrophy in humans [67]. Previous studies have demonstrated that a knockout or deletion of the *CMAH* gene, in mice and humans, respectively, is associated with an aggravation of the DMD phenotype [68], [69]. In cats, the CMAH gene product’s enzymatic activity plays a crucial role in the formation of blood groups. In some cat breeds, such as Burmese, Siamese and Oriental shorthair, certain blood groups (type B) are not present. Consequently, it is possible that in such breeds, the DMD phenotype may be unexpressed or less severe due to the absence of this blood group. How these variants should be reported in a standardized manner is up for debate but at the moment it is the responsibility of the scientific community and the treating physician to be vigilant.

## 5 Limitations

WES has limitations, particularly in detecting structural variants (e.g., copy number variants) and epigenetic modifications, which require complementary methods [70], [71]. Additionally, thorough phenotyping remains essential to accurately interpret sequencing results [72]. Consequently, to be able to match sequencing results with the complaints/symptoms of the patient, a thorough workup of the clinical phenotype should be performed prior to drawing conclusions and setting up a treatment plan [73]. It is also important to note that the performance metrics per phenotype-associated variant and per sample can be influenced by factors specific to the sequencing setup used in this study. Variables such as DNA library quality, sequencing platform, and the number of samples per sequencing lane all contribute to these values. While our findings provide a valuable reference point for assessing WES performance in a clinical veterinary setting, they should be interpreted with consideration of these technical variables, as different laboratories may observe variations depending on their protocols and infrastructure.

A last limitation, specific to this study, is the absence of longitudinal phenotype data for the dogs analyzed. This lack of information prevents us from confirming if symptoms presented itself, particularly for late-onset diseases.

## 6 Conclusion

While WES is well-established in human diagnostics, its application in clinical genetics in veterinary medicine is limited [74]. Our study demonstrates that WES performs well in detecting phenotype-associated variants within target regions, even for newly discovered variants. These findings suggest that WES could be a valuable tool for veterinary diagnostics and screening. However, challenges remain, including the detection of structural variants, the need for standardized clinical reporting, and the importance of thorough phenotyping. One of the first big hurdles that recently was taken, was the development and publication of the Animal Variant Classification Guidelines [75], [76]. Further research and international collaboration are essential to establish guidelines for clinical implementation.

## Ethics declarations

EDTA-blood samples were collected for research purposes. Approval was granted by the local ethical (Faculty of Veterinary Medicine, Ghent University, Belgium) and deontological (Federal Public Service Health, Food Chain Safety and Environment, Brussels, Belgium) committees (EC2017/86). All experiments were carried out in accordance with the approved guidelines.

## Funding

This work was funded by Bijzonder Onderzoeksfonds (10.13039/501100007229BOF) starting Grant (01N04119). The project was carried out with the support of the Fund Albert & Oscar, managed by the 10.13039/501100006282King Baudouin Foundation (https:// kbs-frb.be/en/).

## CRediT authorship contribution statement

**Van Nieuwerburgh Filip:** Writing – review & editing. **Peelman Luc:** Writing – review & editing. **Vossaert Liesbeth:** Writing – review & editing. **Bogaerts Evelien:** Writing – review & editing, Methodology, Conceptualization. **Broeckx Bart J. G.:** Writing – review & editing, Supervision, Resources, Project administration, Funding acquisition, Conceptualization. **Saunders Jimmy H.:** Writing – review & editing. **Boeykens Fréderique:** Writing – original draft, Methodology, Formal analysis, Data curation, Conceptualization.

## Declaration of Competing Interest

The authors declare that there are no conflicts of interest associated with this work.
